# The Schistosomiasis Clinical Trials Landscape: A Systematic Review of Antischistosomal Treatment Efficacy Studies and a Case for Sharing Individual Participant-Level Data (IPD)

**DOI:** 10.1371/journal.pntd.0004784

**Published:** 2016-06-27

**Authors:** Amélie M. Julé, Michel Vaillant, Trudie A. Lang, Philippe J. Guérin, Piero L. Olliaro

**Affiliations:** 1 The Global Heath Network, University of Oxford, Oxford, United Kingdom; 2 Centre for Global Health and Tropical Medicine, Nuffield Department of Medicine, University of Oxford, Oxford, United Kingdom; 3 Competence Centre for Methodology and Statistics (CCMS), Luxembourg Institute of Health, Luxembourg, Luxembourg; 4 WorldWide Antimalarial Resistance Network (WWARN), University of Oxford, Oxford, United Kingdom; 5 UNICEF/UNDP/World Bank/WHO Special Programme on Research and Training in Tropical Diseases (TDR), Geneva, Switzerland; The George Washington University School of Medicine and Health Sciences, UNITED STATES

## Abstract

**Background:**

Schistosomiasis control mainly relies on preventive chemotherapy with praziquantel (PZQ) distributed through mass drug administration. With a target of 260 million treatments yearly, reliably assessing and monitoring efficacy is all-important. Recommendations for treatment and control of schistosomiasis are supported by systematic reviews and meta-analyses of aggregated data, which however also point to limitations due to heterogeneity in trial design, analyses and reporting. Some such limitations could be corrected through access to individual participant-level data (IPD), which facilitates standardised analyses.

**Methodology:**

A systematic literature review was conducted to identify antischistosomal drug efficacy studies performed since 2000; including electronic searches of the Cochrane Infectious Diseases Group specialised register and the Cochrane Library, PubMed, CENTRAL and Embase; complemented with a manual search for articles listed in past reviews. Antischistosomal treatment studies with assessment of outcome within 60 days post-treatment were eligible. Meta-data, i.e. study-level characteristics (*Schistosoma* species, number of patients, drug administered, country, etc.) and efficacy parameters were extracted from published documents to evaluate the scope of an individual-level data sharing platform.

**Principal findings:**

Out of 914 documents screened, 90 studies from 26 countries were included, enrolling 20,517 participants infected with *Schistosoma spp*. and treated with different PZQ regimens or other drugs. Methodologies varied in terms of diagnostic approaches (number of samples and test repeats), time of outcome assessment, and outcome measure (cure rate or egg reduction rate, as an arithmetic or geometric mean), making direct comparison of published data difficult.

**Conclusions:**

This review describes the landscape of schistosomiasis clinical research. The volume of data and the methodological and reporting heterogeneity identified all indicate that there is scope for an individual participant-level database, to allow for standardised analyses.

## Author Summary

Schistosomiasis is a parasitic disease prevalent in tropical climates, especially Africa. To control morbidity and eventually eliminate the disease, the World Health Organisation recommends that populations living in endemic areas be preventively treated with praziquantel. This means hundreds of million treatments over time, which calls for careful monitoring of drug efficacy. Meta-analyses of aggregated data from clinical trials support this policy, but conclusions are limited because studies use diverse methodologies, and detailed analyses (e.g. by age-groups) are not possible. Access to primary data generated by trials would strengthen the evidence and allow answering key questions, such as what the optimum paediatric dose is. This review assesses the number of trials conducted on antischistosomal drugs and their characteristics (where studies were conducted, in which population, what drug(s)/dose(s) were administered, and the methodologies applied), to determine the merit and scope of a common database of antischistosomal efficacy data.

## Introduction

Despite its heavy burden worldwide, schistosomiasis remains a neglected tropical disease (NTD) which fails to attract enough attention from research and funding bodies to generate novel drugs and diagnostics [1,2]. Currently, only one drug, praziquantel (PZQ), is available to treat schistosomiasis [3]. The World Health Organization (WHO) strategic plan for schistosomiasis control aims at controlling morbidity due to schistosomiasis by 2020; eliminating schistosomiasis as a public health problem by 2025; and interrupting transmission of schistosomiasis in selected countries in Africa and all other countries in the rest of the world by 2025 [4]. To reduce morbidity and transmission, the WHO recommends the use of PZQ, given as preventive chemotherapy to endemic populations [3]. Along with evidence from pre-clinical studies [5,6] and individual clinical trials, the efficacy and safety of PZQ is further supported by systematic reviews and meta-analyses of clinical trials [7,8]. However, aggregated data meta-analysis suffers from the heterogeneity in the design and reporting of the published studies [9,10]. This lack of standardisation is a common problem across several NTDs; specifically, in the case of schistosomiasis, it concerns diagnosis and test of cure (diagnostic tests tend to be performed on variable numbers of samples per individual), outcome measures (cure rate (CR), or egg reduction rate (ERR) calculated by arithmetic or geometric mean), and time of assessment [11]. Access to detailed, individual-level participant data (IPD) for meta-analyses would circumvent some of those limitations and allow harmonisation of relevant variables and analyses [9,10], thus enabling more meaningful analyses and comparisons across individuals or studies. This could unravel yet unknown aspects of drug efficacy and safety, in specific populations and over time. Such a model for systematic review and secondary use of clinical trials’ data has brought valuable evidence for other disease areas, whether non-communicable (as reviewed in [10]) or infectious (e.g. malaria [12]).

Monitoring PZQ efficacy is crucial, considering the size of the problem and the inherent risks of resistance [13]. In 2013, of the some 260 million people requiring treatment for schistosomiasis (of whom, 46% school-aged children), 13% did receive preventive chemotherapy with PZQ. While still far from the intended programme target, this is close to 40 million treatments (covering two thirds of the school-aged children at need) [14].

Recognising the potential benefits of a shared IPD database, the UNICEF/UNDP/World Bank/WHO Special Programme on Research and Training in Tropical Diseases (TDR) and the WHO programme for NTDs are currently working with a range of stakeholders to build a dedicated, sustainable data-sharing platform for antischistosomal efficacy studies [15]. This initiative also aims to respond to growing requirements for researchers to share their data, coming notably from funding bodies, journal editors, WHO and to some extent, the public [16–21].

The present work was performed to lay the groundwork for the development of the schistosomiasis data-sharing platform, and had three main objectives. Firstly, we sought to identify efficacy studies of antischistosomal drugs performed since 2000, and published between January 2001 and May 2015 (either as an article or as a conference abstract). We followed PRISMA guidelines [22] (S1 Checklist), so that the resulting list of studies could form the basis for future collection and meta-analyses of IPD. Focus on recent studies is for practical reasons (datasets less likely lost, corrupted or unreadable), but we would encourage investigators to share data from earlier studies too, if available. Secondly, we aimed to assess the volume of such IPD and its inherent characteristics, e.g. species, treatment, participants’ age-group, and study site. Such an assessment gives an overview of the expected range of data to be potentially assembled, and can help design the standardised database by highlighting key variables and meta-data to consider in its structure. Thirdly, we investigated further the heterogeneity in study design and methodologies that has already been highlighted by past systematic reviews and aggregated-data meta-analyses [11].

## Methods

### Literature search

To identify relevant references, the following journal databases were interrogated: the Cochrane Infectious Diseases Group specialised register and the Cochrane Library, PubMed, CENTRAL and Embase. Bibliographic references of previous meta-analyses of antischistosomal trials [7,8,11,23,24] were also manually examined. No restriction on publication language was used. Where possible, search results were filtered for ‘human’ as the species of study.

Keywords belonged to three main themes reflecting the purpose of the search: (1) schistosomiasis [(1.1.) schisto*; (1.2.) ‘schistosoma hematobium’ OR ‘schistosomiasis haematobia’ OR ‘schistosom* haematobi*‘ OR ‘urinary schistosom*’ OR ‘urogenital schistosom*’; (1.3.) ‘schistosoma japonicum’ OR ‘schistosomiasis japonica’ OR ‘schistosom* japonic*’ OR ‘oriental schistosom*‘; (1.4.) ‘schistosoma mansoni’ OR ‘schistosomiasis mansoni’ OR ‘schistosom* mansoni’ OR ‘intestinal schistosom*’ OR ‘bilharz*’]; (2) clinical study [treatment OR trial OR random* OR double-blind* OR cohort OR prospective]; and (3) specific drugs [(3.1.) praziquantel OR artesunate OR artemether OR metrifonate OR albendazole; (3.2.) oxamniquine OR mefloquine].

Of note, although albendazole has not shown efficacy against *Schistosoma spp*., it was included in the keywords for search in order to better identify studies performed by the soil-transmitted helminthiases (STHs) community which may have looked at schistosomiasis aside of their major study.

Four subsequent searches were performed between 25 May 2015 and 12 June 2015. The three first focussed on one major pathogenic species of *Schistosoma* each (*S*. *haematobium*, *S*. *japonicum* and *S*. *mansoni*), and thus used keywords (1.2., 1.3. or 1.4.) AND (2) AND (3.1.). As they constituted an update of previous (published) Cochrane systematic searches, the two searches for literature on *S*. *haematobium* and *S*. *mansoni* were limited to records published from 2000 up to the date of search (25 May 2015). The search for *S*. *japonicum* used no such restriction on publication date, as no prior Cochrane search had been performed. The fourth, complementary search looked at all species altogether and was restricted to publications from 2001, but focussed on different drugs that were missing in the initial searches, therefore combining keywords (1.1.) AND (2) AND (3.2.).

### Eligibility screening

Only abstracts published from 2001, and whenever necessary, the corresponding full texts, were eventually screened for eligibility. In line with the primary objective of estimating the size of a global database of antischistosomal efficacy data, and as the likelihood of retrieving datasets dating back before 2000 is much more limited [25], it was decided to exclude articles reporting on studies that had been completed before 1 January 2000. Abstracts of conferences run before 1 January 2014 were also excluded, owing to the difficulty in establishing whether earlier abstracts were duplicate records of articles published within the considered time period. Finally, all case reports or case series, as well as non-primary research studies (meta-analyses, reviews, textbook chapters, opinion papers and comments) were excluded.

Regardless of their design and associated risk of bias, remaining studies where antischistosomal (registered or experimental) drugs had been administered to human subjects in an endemic setting were eligible, provided that: (i) at least a subset of participants was positively diagnosed for infection with at least one of the three *Schistosoma* species of interest, prior to receiving treatment; and (ii) a post-treatment diagnostic test was performed on the same participants to assess outcome within 60 days, where day 0 is the day of administration of the first (and in most cases, single) treatment dose.

The WHO recommends assessing the outcome within 21 days of therapy, in order to distinguish between cases of treatment failure and cases of early reinfection [26]. However, the maturation time of *Schistosoma* worms in the human host induces a delay of at least 4–5 weeks between the moment of infection and the excretion of eggs [27]. Within the 60 days post-treatment window, individuals who were initially cured but got re-infected are still likely to excrete low numbers of eggs (if at all), and to appear negative upon diagnosis by egg count. The 60-day limit was thus retained, to avoid excluding too many efficacy studies which performed an outcome assessment beyond the recommended 21-day timeframe.

### Data extraction and analysis

Relevant information from eligible studies published in English or French was extracted by one researcher, using a detailed variable dictionary to facilitate consistent extraction. Help from co-authors and from native Chinese speakers was sought, as required. Extracted information was recorded using a spreadsheet document (Microsoft Excel 2000), and analysed using R (version 3.1.3) [28]. The source data used for analysis, the associated dictionary and the R code are all available as supporting documentation in S1 Dataset (Zip).

### Estimation of the number of participants

Some of the eligible studies had a broad scope, and were looking simultaneously at schistosomiasis and at other parasitic infections, in particular STHs. Articles on such studies did not systematically report on the exact number of participants with a *Schistosoma* infection at baseline, thus the need to estimate this number for the purpose of this review.

Whenever possible, the number of *Schistosoma*-positive participants was re-calculated from the specified prevalence of infection among enrolled participants. If the prevalence amongst enrolled participants was unknown, the reported prevalence in the study area was used instead. Finally, in the rare case when there was no mention of prevalence at all, half of the cohort was arbitrarily considered infected with *Schistosoma spp*. at baseline (i.e. assumed prevalence of 50% among enrolled participants).

Of note and unless more specific information was provided in the article, the retained frequency of infection (exact prevalence in study population, reported prevalence in study area, or 0.5) was then consistently applied as a correction factor when estimating the number of relevant participants in subgroups (e.g. treatment group, group followed-up at later time-points): this implies that the subgroup characteristic and the initial infection status of the participant would be independent.

### Quality control

After extraction and coding of all information as per the variable dictionary, the datasets were checked for potential errors. To do so, each article was systematically re-examined for variables considered most ‘impactful’. These were primarily the variables recording numbers of relevant participants (*Schistosoma*-positive at baseline, and who successfully received the assigned treatment regimen) at both enrolment and follow-up, and in each assignment arm; because those data directly impact on the total number of IPD, whose estimation was our primary objective. The species and drug(s) under study were also double-checked, as the information coded in those variables was used to filter articles by category (*S*. *haematobium* vs. *S*. *mansoni*, PZQ vs. other drugs, etc.) and to create specific datasets for analyses by sub-category. Finally, impactful variables also included the time-points of post-treatment assessment of outcome, as they influenced inclusion/exclusion of the article from the analysis.

This double-checking led to the correction of 3 to 12 errors per variable, mainly due to ambiguity in the original articles (e.g. unclear flowchart of included, treated and followed-up participants), and their correction did not significantly change the total of estimated IPD.

A subset of articles was also randomly selected for full, independent re-analysis. This step unravelled 3 typos, which, spread across a total of 45 variables (excluding the core variables previously double-checked) and 12 articles, suggest an error rate of 0.56% in the data as a whole, and of >1% per variable.

## Results

### Search results, number of eligible studies and corresponding IPD

**Fig 1:**
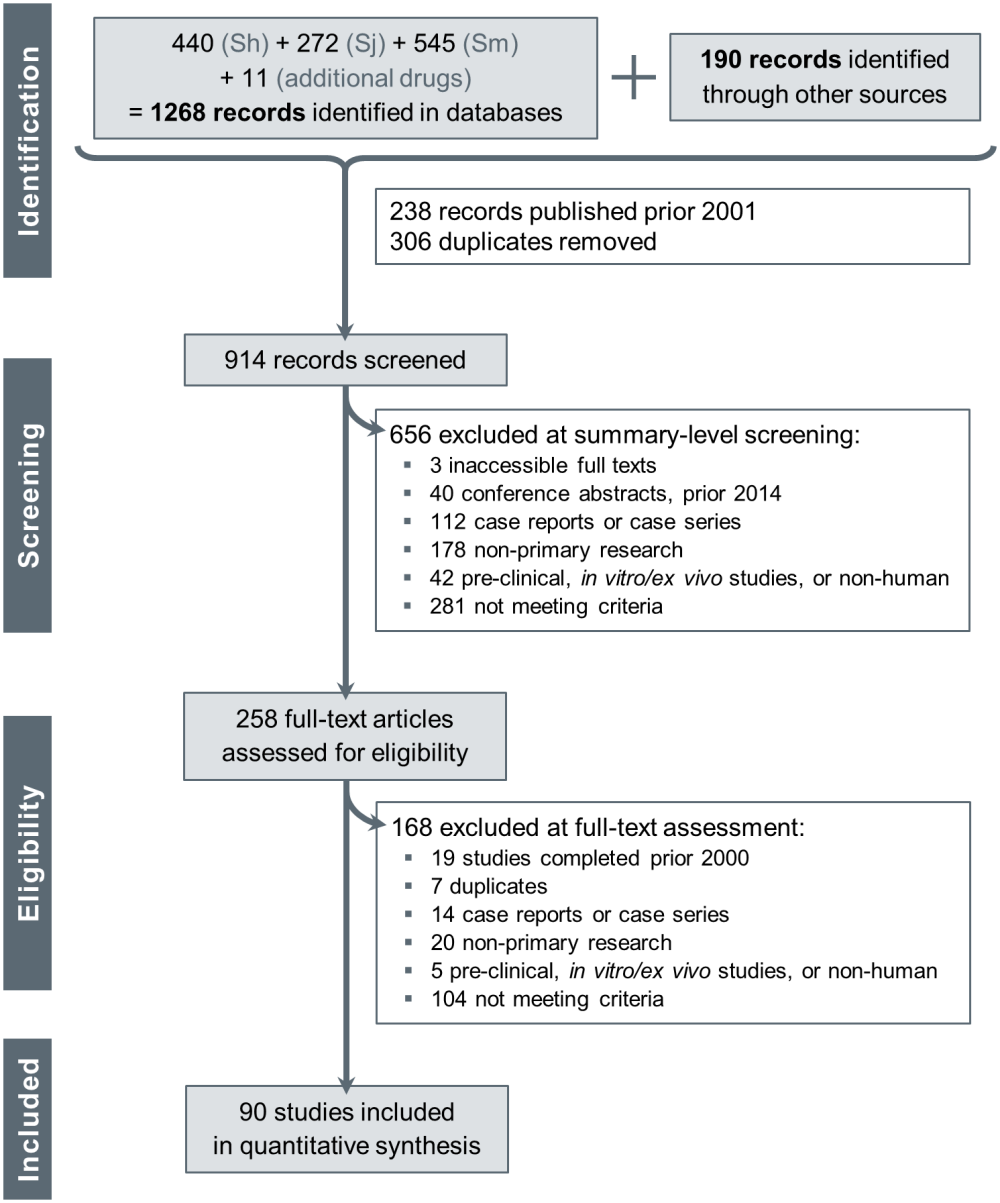
PRISMA diagram. Flow of the study references from identification during the search, through screening and eligibility and up until inclusion. The search strategy and eligibility criteria are detailed in the methods section. Briefly, all clinical studies of antischistosomal drugs, completed in 2000 or later and performed in an endemic setting, were included if at least some of the recruited participants were diagnosed for infection with *Schistosoma spp*. before and within 60 days after treatment.

The three species-specific searches of the literature in electronic resources yielded 545, 440 and 272 study abstracts for *S*. *mansoni*, *S*. *haematobium* and *S*. *japonicum*, respectively. The later search for additional drugs gave only 11 additional results, of which 6 abstracts reported on studies of *S*. *mansoni*, and 5 on *S*. *haematobium*. In parallel, 190 references were identified in the bibliography of published meta-analyses [7,8,11,23,24]. Overall, those amounted to 914 unique abstracts published since 1 January 2001, of which 90 studies were retained after eligibility screening (Fig 1).

Although most studies could be thoroughly evaluated, the case of three abstracts [29–31] remained unclear, and we could not access the corresponding article. In addition, the eligibility of two other studies [32,33] could not be ascertained despite access to full text. At this stage, and as this review is a preliminary phase prior to possible collection of IPD and performance of clinically relevant meta-analyses, no attempt was made to contact the authors and clarify missing information. This step shall be performed later, along with a public call addressed to the schistosomiasis community to help identify more studies that might have been missed by this scoping review (e.g. studies that were not published, or published in non-indexed journals).

The 90 included studies correspond to 104 cohorts, where a ‘cohort’ is the largest possible subset of study participants from the same country, which followed the same protocol and thus share the same study-level characteristics (except for the assigned intervention in multi-arm studies). Most studies (79) involved a single cohort, but some looked at different groups such as infants and adults who were followed up at different time-points, or involved sites across several countries with different parasite species and diagnostic approaches. Those study groups were thus considered to be separate cohorts, and the analysis by cohort is preferred whenever comparing the specific methodologies and protocols applied.

The 90 studies enrolled an estimated total of 20,517 participants of relevance for schistosomiasis treatment efficacy meta-analyses. One conference abstract did not provide any information to estimate the number of participants: accordingly, this record did not contribute to analyses by number of participants, but was still considered for analyses by cohort or study. Throughout this article, whenever mentioning a number of participants, it is derived from the number of enrolled participants infected with *Schistosoma spp*. at baseline, and who received the full treatment regimen they were assigned. The number of participants refers to the initial cohorts, as losses to follow-up were inconsistently reported and therefore could not be accounted for here (estimates suggest that the total of participants with complete data is about 17,500).

### IPD characteristics of relevance for clinical or epidemiological analyses

**Fig 2:**
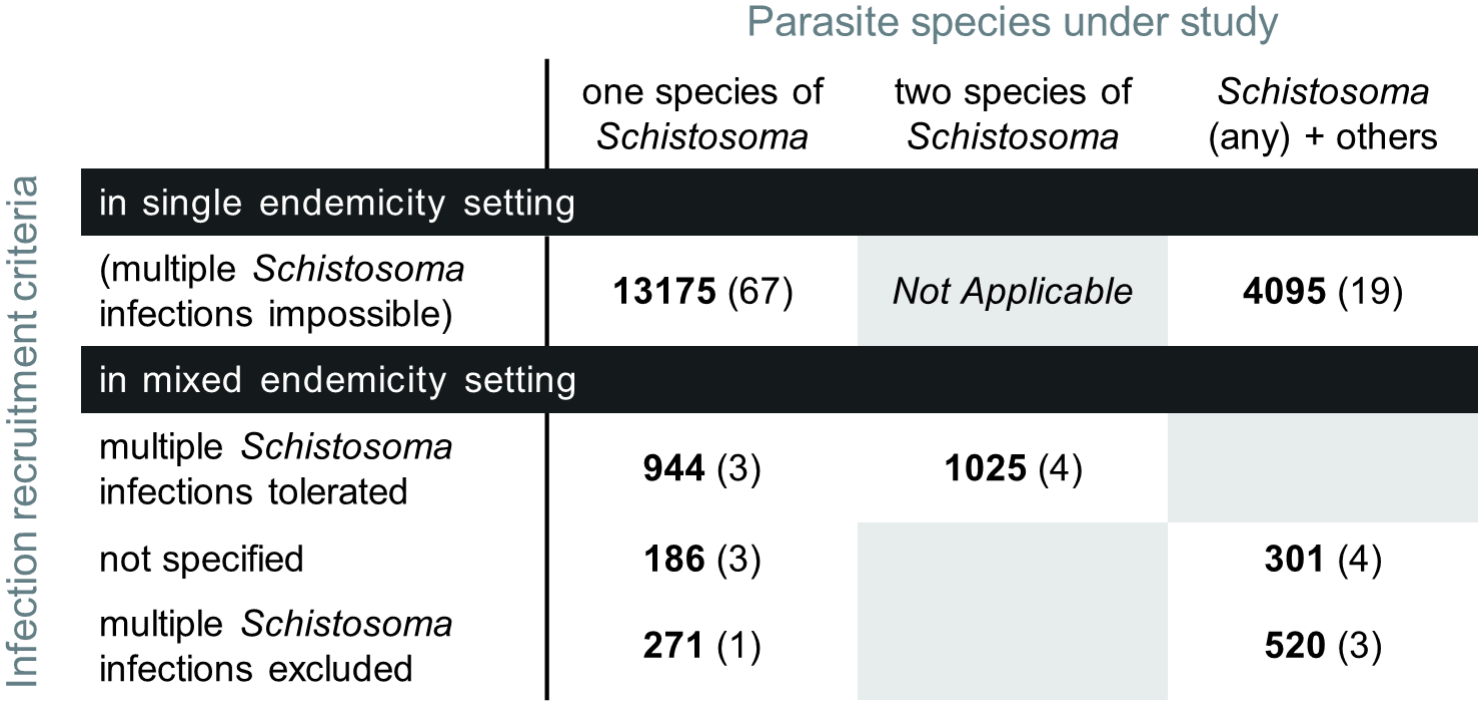
Type of *Schistosoma* infection required for recruitment in the study, depending on the endemic setting and parasite of interest. Numbers in bold are totals of participants (out of 20,517) recruited in the specified setting (single or mixed endemicity), applying the corresponding criteria (multiple *Schistosoma* infections tolerated or strictly excluded) and for the study of the corresponding parasite(s) (one or two *Schistosoma* species, and possibly other parasites as well). Numbers in brackets are corresponding totals of cohorts (out of 104).

A major advantage of statistical meta-analyses based on IPD pooled from multiple studies is to provide large numbers. With this method, specific sub-populations whose outcomes are usually ‘hidden’ in aggregated data could be individually considered for eligibility in re-analyses on a case-by-case basis, depending on the objectives. Here for instance, 26 cohorts are formed of participants infected with several possible parasites, which made it difficult to extract information on *Schistosoma*-infected participants using published information only (Fig 2). Depending on the purpose of the meta-analysis, it could be interesting to retrieve data about these estimated 4,916 participants infected with schistosomiasis, and treated with drugs having antischistosomal activity as part of larger studies focussing on other parasites (Fig 2).

**Fig 3:**
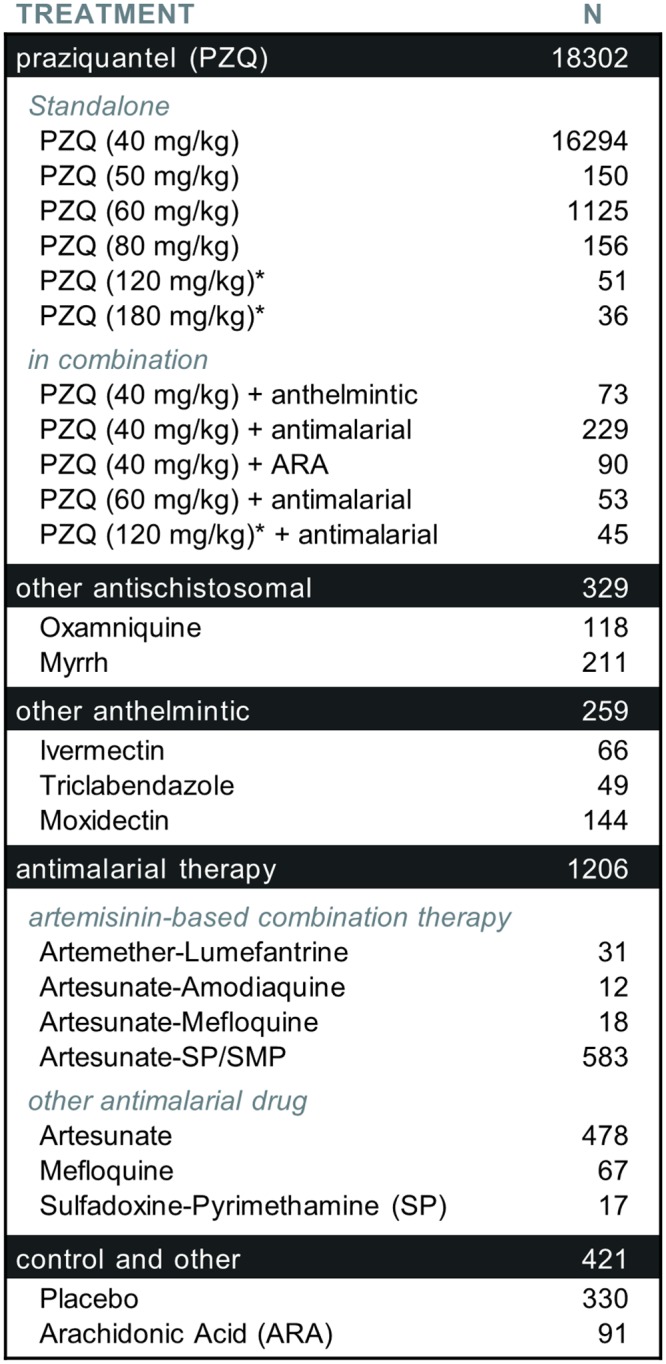
Antischistosomal treatments tested in the included studies. N: Number of participants, infected with *Schistosoma spp*. at baseline, and who successfully received the complete assigned regimen. * Treatment dose delivered in split doses of 20 mg/kg every 4 hours (PZQ 120 mg/kg), or 60 mg/kg every 24 hours (PZQ 180 mg/kg).

**Fig 4:**
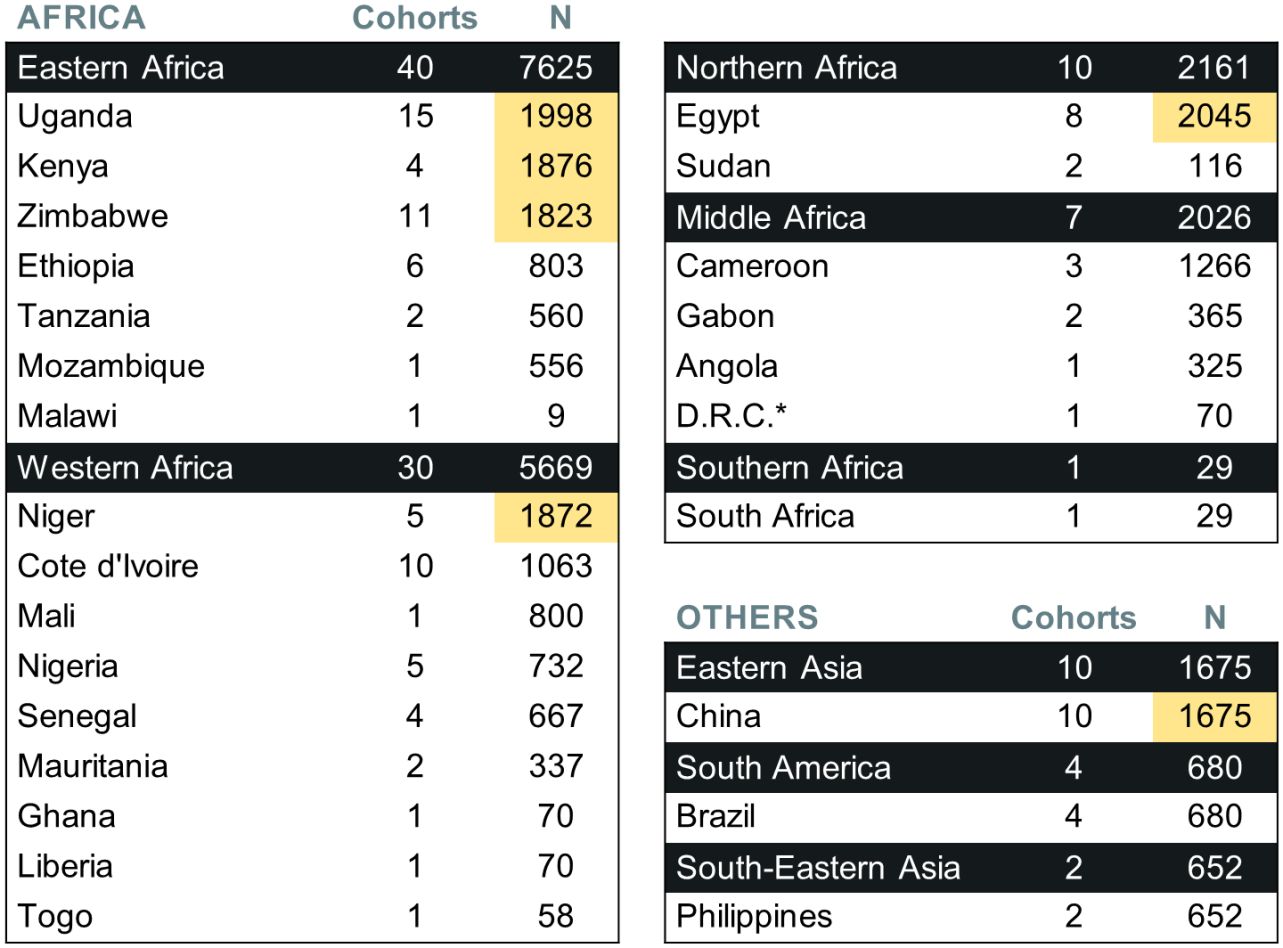
Number of cohorts and corresponding participants recruited by country. Country names and world macro-regions are based on the United Nations Statistics Division (UN data) classification [34]. Countries that account for more than 8% of the total IPD are highlighted in yellow. N: Number of participants recruited. * D.R.C.: Democratic Republic of the Congo.

**Fig 5:**
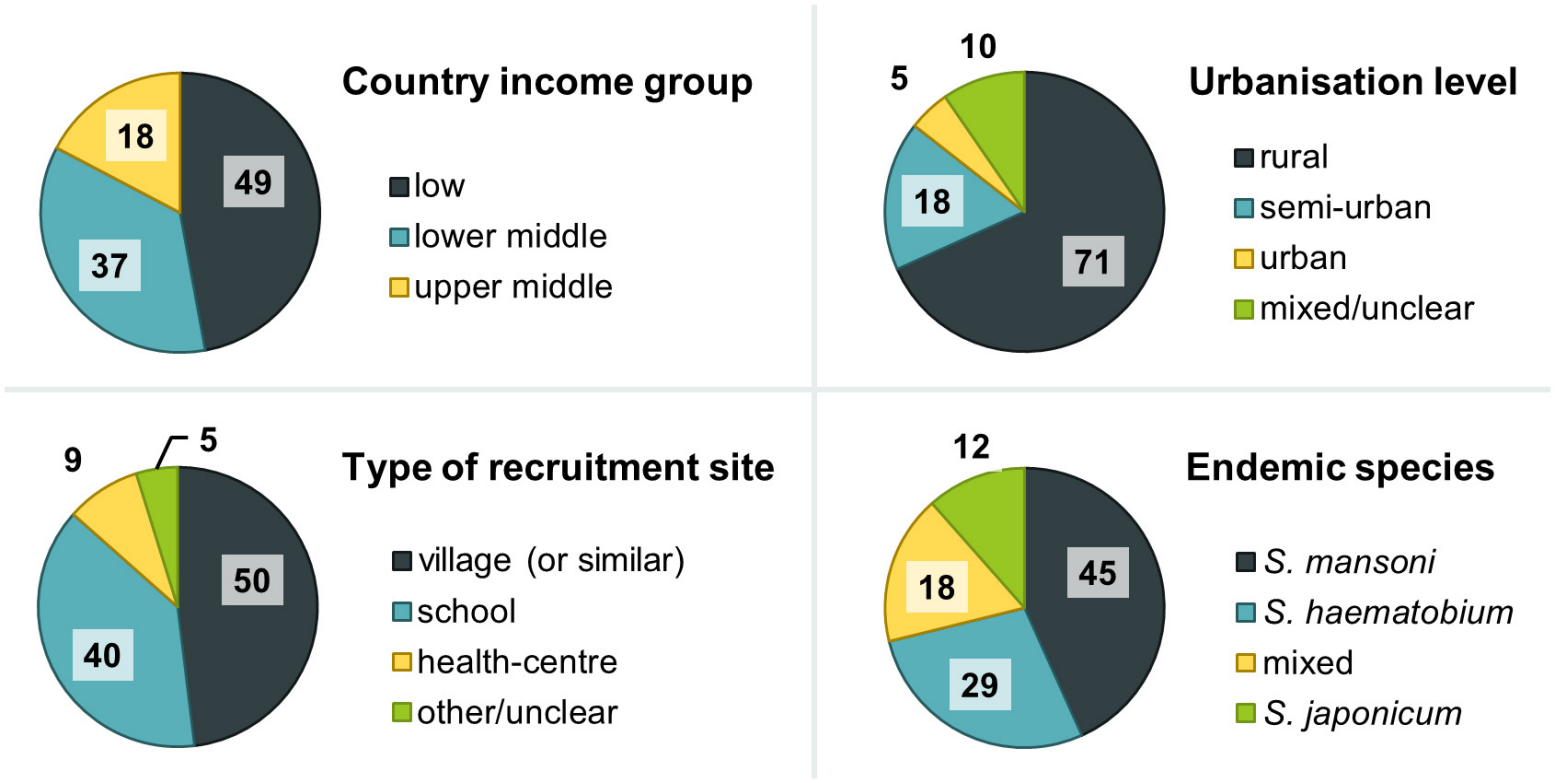
Variety of settings in which cohorts were recruited. Counts correspond to the number of cohorts (total per pie chart, n = 104) recruited at a site with the corresponding characteristic. Country income groups are based on World Bank classification [35].

**Fig 6:**
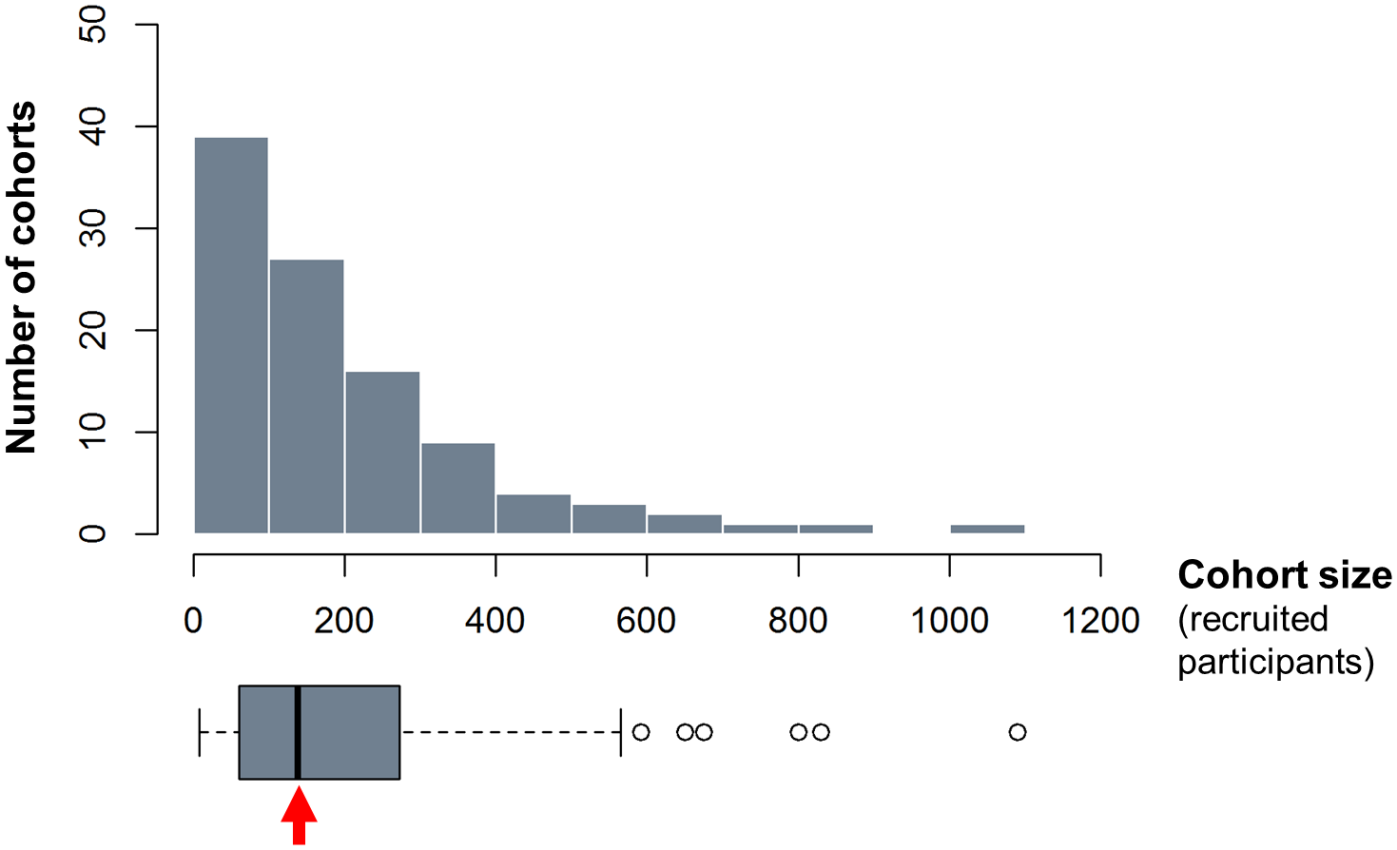
Cohort size distribution. 103 cohorts contribute to the histogram, as the number of recruited participants remains unknown for one cohort. The box covers values ranging between the first (60.5 participants) and third (273 participants) quartiles. The red arrow points to the median cohort size (138 participants). Whiskers extend to values up to 1.5 times the interquartile range; dots show outliers (up to a maximum of 1,090 participants).

The 104 cohorts were also recruited in varied endemic settings and countries, and tested several therapies—mostly various doses and regimens of PZQ, but also a dozen other therapies (Fig 3). Considering the large amount of participants having received PZQ at the dose of 40 mg/kg of body weight (the WHO-recommended regimen for preventive chemotherapy [3]), pooled data could help better characterize responses and identify subgroups with sub-optimal responses, and evaluate efficacy over time and in different settings (the 104 cohorts were recruited in 26 different countries (Fig 4) and in various contexts (Fig 5)). In addition, the 20,517 IPD are likely to represent different sub-populations of interest and that remain under-studied, such as preschool-aged children (30 of the 104 cohorts clearly recruited at least one participant aged strictly less than 6 years), or pregnant women (2 cohorts focussed on this group, i.e. a total of 452 participants and possibly more, as several cohorts seemed not to specifically exclude those women). Most cohorts were relatively small, with 62.5% of them involving less than 200 participants with schistosomiasis at baseline (Fig 6), thus the need to pool data together to reach the sample size giving enough statistical power to unravel drug effects in those smaller populations.

### Heterogeneity in study design hampering meta-analyses of aggregated data

**Fig 7:**
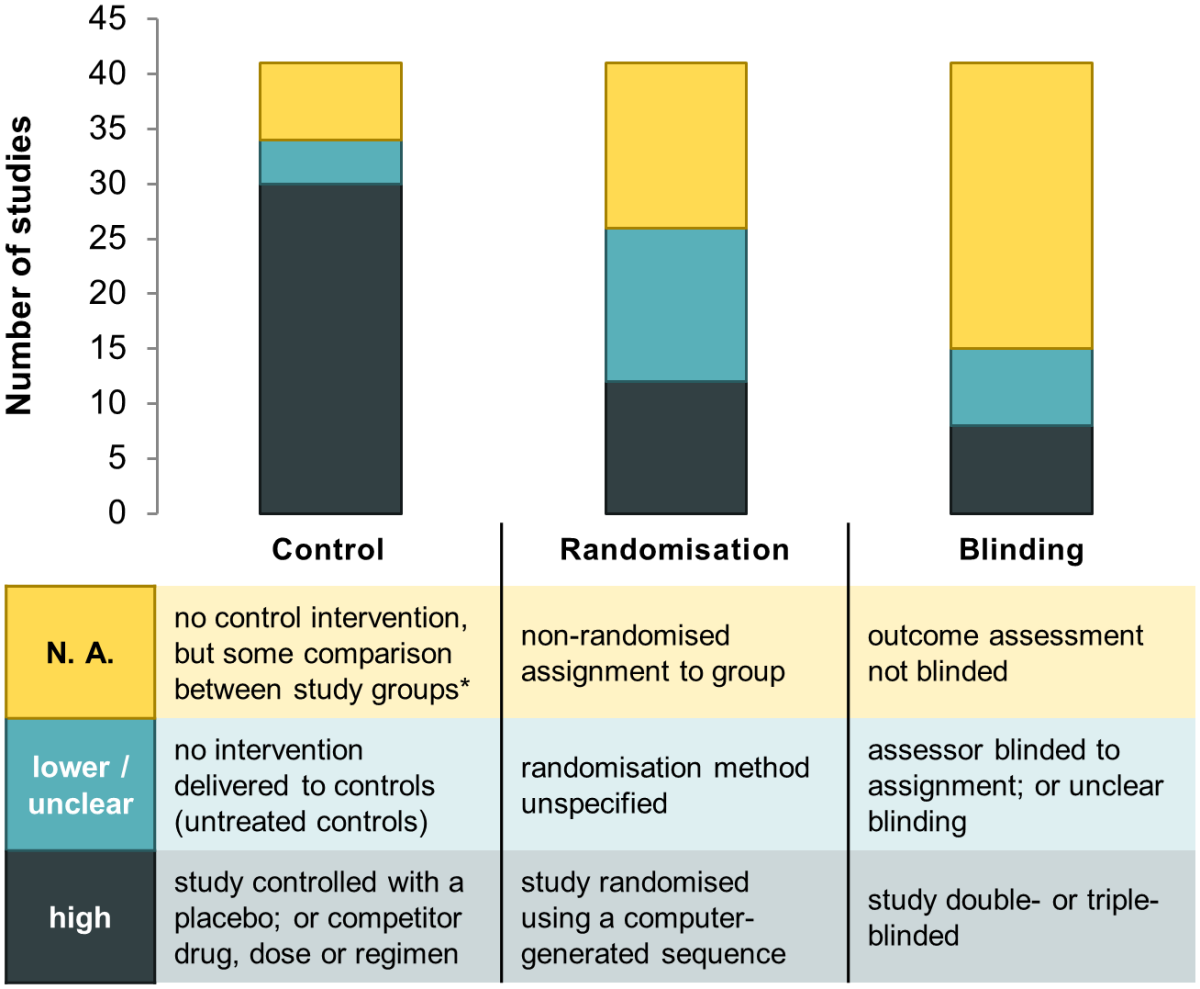
Characteristics of the study design among multiple-arm studies (n = 41), with quality assessment where applicable. Independently for each of the three design elements (control, randomisation and blinding), the quality as compared to expected ‘gold standards’ of treatment efficacy trials was estimated as ‘high’, ‘lower/unclear’, or ‘N.A.’ (not applicable), as detailed in the table accompanying the graph. * For example, comparison of response to the same treatment within different endemic settings.

Not all of the studies included in the present review would meet more stringent eligibility criteria as required for inclusion in Cochrane systematic reviews [36]. No judgement on the type of study design was performed prior to inclusion. Potentially all good-quality drug efficacy data, even if not obtained from a randomised controlled trial (RCT), can contribute to IPD meta-analyses (comparison of continued drug efficacy in specific areas, modelling and model validation, trial methodology questions, etc.), and thus should be eligible for inclusion in a global database. Less than half (41) of the studies included here were comparative, and a third of them (30 studies) delivered a comparator intervention to the control arm (Fig 7). Of these comparative studies, 26 studies were randomised, out of which 12 mentioned a computer-generated randomisation sequence (Fig 7). Access to IPD would facilitate the appraisal of study design, for example by enabling to check the reliability of the randomisation method [9,10].

**Fig 8:**
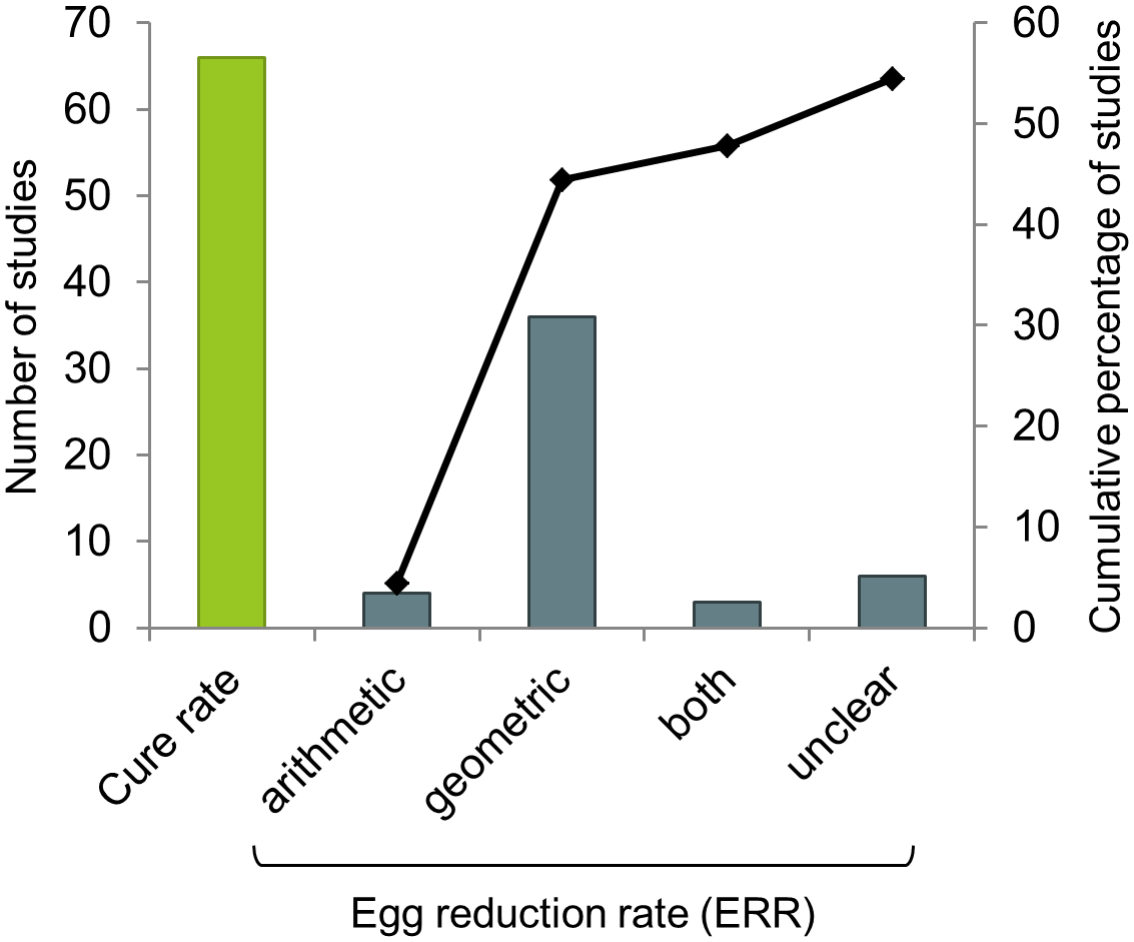
Outcome measure reported in published article or conference abstract. The histogram bars express the total of studies reporting on the primary outcome using the corresponding computation (CR or ERR expressed as arithmetic, geometric mean or both). The curve expresses the cumulative percentage of studies reporting an ERR. All studies reporting an ERR also reported a CR, except for one.

Owing to those loose eligibility criteria regarding study design, the studies included in the present review did not necessarily have the assessment of efficacy as their primary objective. Some rather focussed on specific immune responses following drug administration (15 studies), or on the search for specific biomarkers of infection and new approaches to diagnosis (14 studies). This means that not all studies are expected to report a drug efficacy outcome such as a CR or an ERR. The CR, or proportion of participants who were infected at baseline and negative at follow-up [37], was the most frequently reported measure of drug efficacy, with 66 of the 90 studies providing such information (Fig 8).

As the diagnosis of schistosomiasis is mainly performed by counting *Schistosoma* eggs in excreta (urine or stool for urinary and intestinal schistosomiasis, respectively), drug efficacy can also be assessed as ERR, or variation in the mean number of eggs found in the excreta of the population before and after treatment [26]. Studies however tend to compute the ERR in different manners, and in particular, to derive it from an arithmetic or a geometric mean (here 7 and 39 articles, respectively, Fig 8). Such variability in efficacy reporting is a major issue in meta-analysis of aggregated data, which could be solved by recalculating measures using the original IPD, and ideally, using the original raw egg count from each of several measurements done for each participant.

### Other causes of heterogeneity

**Fig 9:**
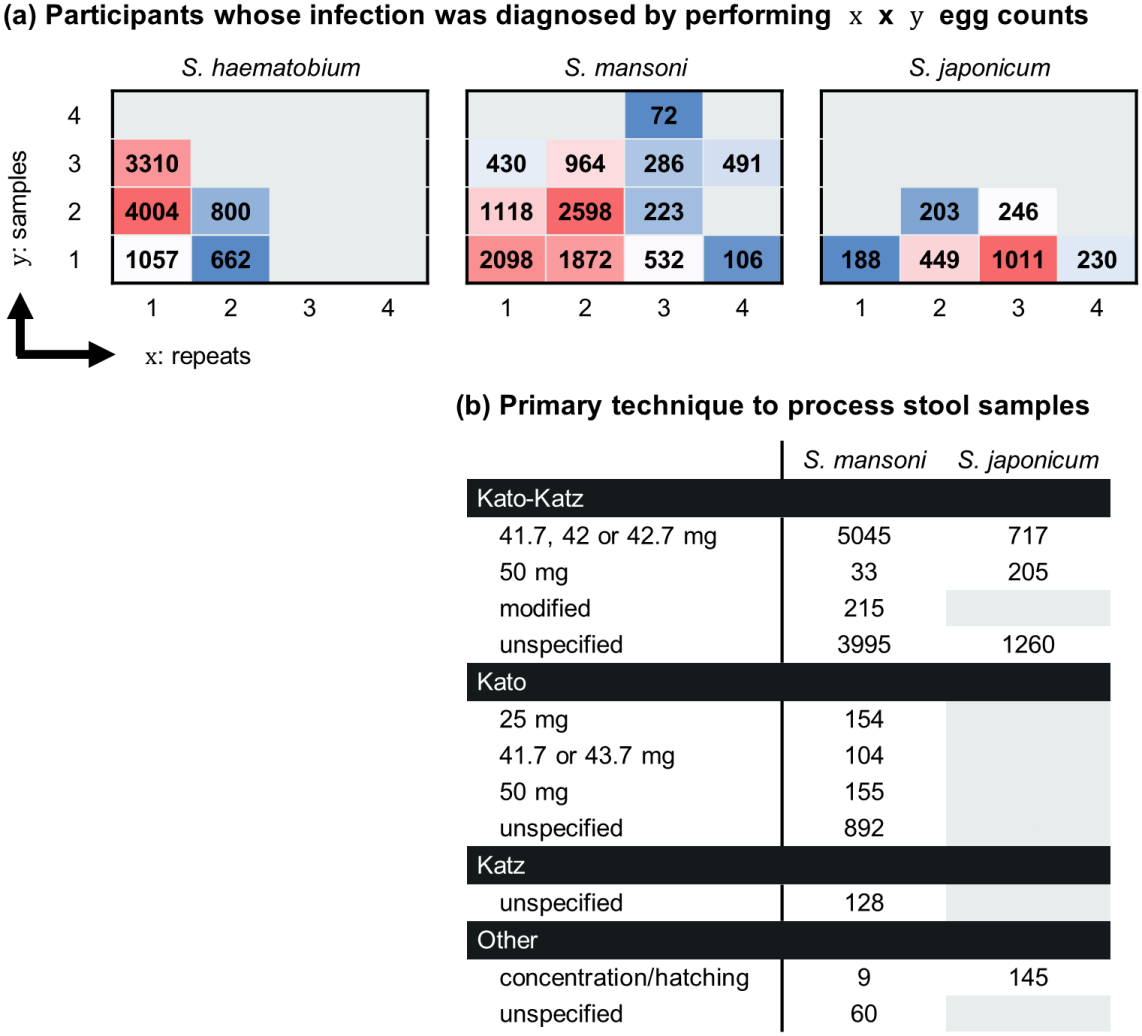
Approach for the diagnosis of infection with *S*. *haematobium*, *S*. *mansoni* or *S*. *japonicum* by egg count. (a) For each species, participants were diagnosed by counting ova excreted in urine (*S*. *haematobium*) or stools (*S*. *mansoni* or *S*. *japonicum*). The diagnostic test was performed on *y* independent samples, and repeated *x* times on each sample. Values are numbers of participants. (b) While preparation of urine samples was standard (10 mL urine filtration and microscopy slide), techniques used to prepare stool samples varied. Values are numbers of participants whose stools were processed using the corresponding technique (total: 10,790 and 2,327 participants for *S*. *mansoni* and *S*. *japonicum*, respectively).

In all cohorts, the primary method to diagnose infection at screening was egg detection and counting in excreta. Eight cohorts also diagnosed participants by quantifying levels of schistosomiasis circulating anodic or cathodic antigen in body fluids (urine or serum). Of those 8 cohorts, 3 used only the latter circulating antigen method to assess the outcome after treatment. The most frequent method to diagnose infection with *Schistosoma spp*. is thus counting eggs; however, differences exist in how samples are prepared (different techniques may be used to process samples prior to egg count under microscopy, especially for the preparation of stools, Fig 9), and how many samples are analysed (numbers of samples and/or test repeats, Fig 9). Since data are reported as means, and the sensitivity of the method depends on the number of independent egg counts for each subject, access to pooled IPD enables better to quantify the extent of this variation in sensitivity, and how it impacts on reported treatment outcomes [24].

**Fig 10:**
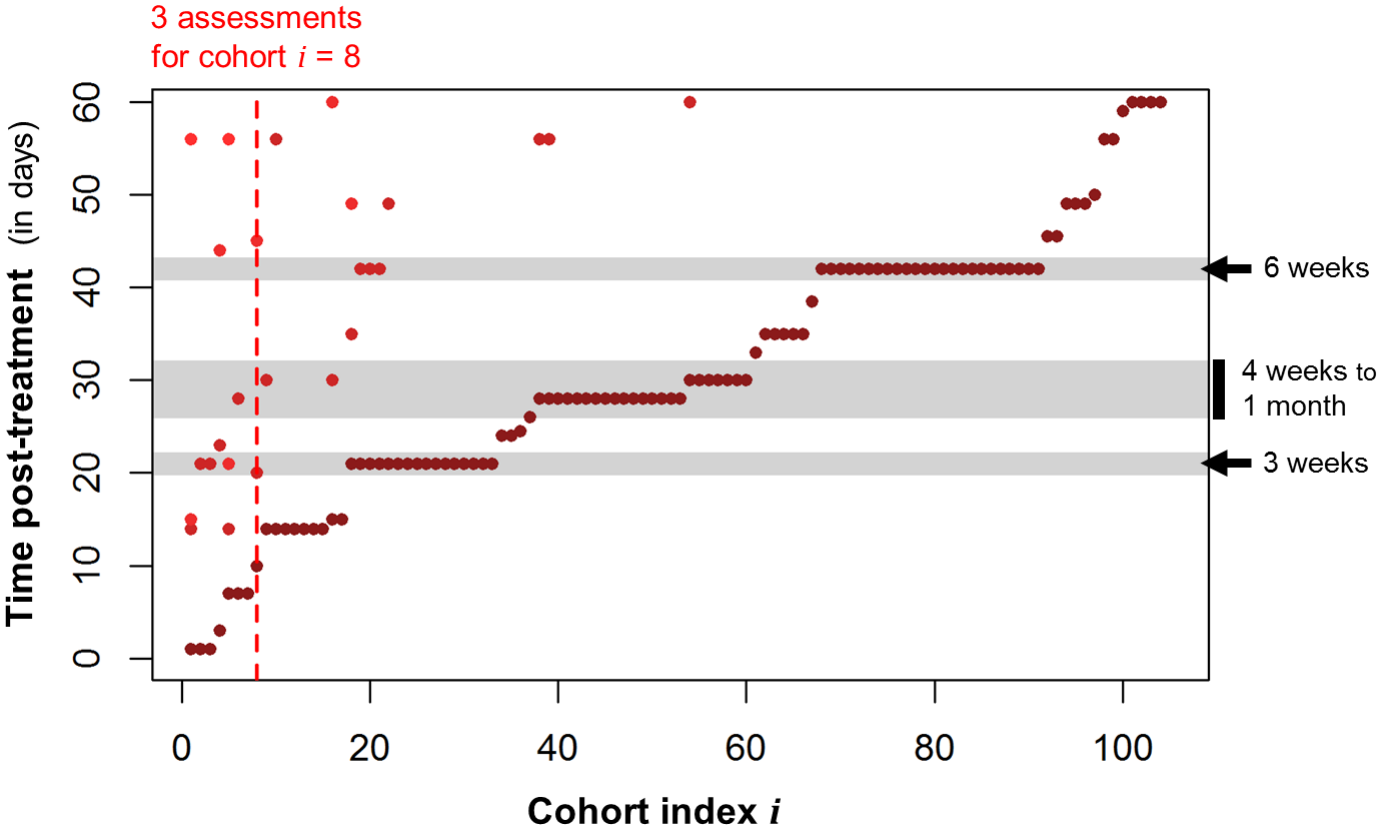
Time of outcome assessment after treatment. For each cohort (indexed from 1 to 104, in increasing order of first time of outcome assessment), all the time-points (within 60 days) at which an assessment of outcome occurred after treatment are represented by one dot. Darker dots: earlier time-point. Lighter dot: later time-point. As an illustration, the dotted line highlights cohort i = 8, for which 3 assessments of outcome were performed at Day 10, 20 and 45. The weekly distribution of the times of outcome assessment seem to depend on species (Fischer’s Exact Test, p-value = 0.01835), with *S*. *haematobium* infection preferably re-assessed at later time-points.

The time at which outcome is assessed post-treatment also varies across studies (Fig 10): 48% of the cohorts (50 cohorts corresponding to 8,144 participants) have at least one data point at 3 to 4 weeks post-treatment, and 20 of those cohorts also have later assessments, sometimes much beyond the analysed 60-day limit. Such cohorts with data points at both 3–4 weeks and later provide opportunities for analyses distinguishing between initial cure and potential reinfection patterns.

## Discussion

This review provides an overview of the landscape of schistosomiasis clinical research, focused on drug efficacy trials conducted over the past 15 years. We identify a total of approximately 20,000 participants enrolled in antischistosomal efficacy studies, covering the three main *Schistosoma* species, and a range of drugs and regimens, study participants, methods (diagnosis and treatment outcomes), countries and contexts. This supports the value of collating data within a global database, as the opportunities for analyses of clinical relevance would be plenty. At best, IPD from schistosomiasis studies should be shared at the level of the raw data, including for instance egg counts for each sample taken.

### Limitations

We are cognisant that not all the data identified here may be available (e.g., electronic dataset non-existent, corrupted or lost), or be made available (some data generators might be reluctant to share) [25,38], but also that more datasets might be available than those captured here. Those include national control programmes surveys (most of which are generally not published), and studies still on-going and/or awaiting publication. The delay between study completion and publication is sometimes very long, ranging from 5 months to 8 years in the case of the articles analysed here (S1 Fig); and only 12 of 90 studies were found to be registered in ClinicalTrials.gov or Controlled-Trials.com. In addition, several studies published locally in languages other than English could also have been missed. No restriction on language was used in the search, but regional registries such as LILACS (South American journals) [39] and CNKI (Chinese journals) [40] were not interrogated. Only 4 of the 90 included articles were in French, and none was in Portuguese or Chinese despite Brazil and China having reputed control programmes and productive research institutes.

Few to no data on oxamniquine (OXA) and metrifonate (MTF) could be identified in the post-2000 timeframe. A search of PubMed articles published in the 1986–2000 period identified at least 2000 and 500 more IPD for OXA and MTF, respectively. Though experience suggests that such ‘older’ data will be more difficult to retrieve [25], efforts to collect past data from Brazil on OXA are under way and should be promoted, in order to provide a more complete picture of the efficacy of several antischistosomal drugs, and its variations over time. Similarly, the collection of data from China should yield additional data on *S*. *japonicum*, which is currently under-represented (2,327 IPD) as compared to *S*. *haematobium* (9,833 IPD) and *S*. *mansoni* (10,790 IPD).

Moreover, local variations in the preferred post-treatment time of outcome assessment may have led to the exclusion of yet relevant studies. When consulted on the development of an antischistosomal data-sharing platform, some investigators suggested to extend the boundary of acceptable time of first assessment to 3 months (90 days), instead of the 2 months (60 days) used as a cut-off for inclusion in the present review: 104 articles were excluded at screening on the grounds of the outcome assessment occurring too late, of which many had performed it at 3 months post-treatment. The preferred timeframe for study inclusion should also depend on the purpose of the subsequent meta-analysis, whether strictly restricted to drug efficacy assessment, or aiming at estimating long-term effects on infection intensity in the population.

### Significance

Despite its limitations, the present review confirms and extends the problem of the variability in clinical research methods and reporting [11], which makes data summaries difficult to re-analyse and undermines the strength of evidence. Some sources of variability could be corrected with access to IPD and application of standardised analyses, as demonstrated by the example of efficacy outcomes: with IPD, it is possible to recalculate ERRs from distinct studies in a homogeneous manner using arithmetic means (as recommended by the WHO [26]), and to explore and compare alternative ways of expressing efficacy, as already done on an initial dataset of up to 4,740 individuals [24,41]. Other sources of variability will be more difficult to correct, but a global database would allow to pool data from studies with consistent diagnostic approaches or, as shown in Olliaro *et al*. [24], to analyse the entire database using the WHO standard operating procedures (one single test of one single sample [26]).

Based on the present analysis and on our past experience of performing IPD meta-analyses [24,41], we find that the following variables would be most useful: site(s) and country of study, *Schistosoma* species under study, year of study start and stop, diagnostic method (number of samples and test repeats, technique of samples’ preparation), regimen (drug name, dose, formulation and treatment regimen, if applicable) and time of follow-up(s). Dates of different events (recruitment, treatment and follow-up(s)) and associated outcomes (e.g. diagnostic result, adverse events) should also be collected for each study participant.

As a final consideration, although 20,000 (and possibly more) IPD is significant and justifies efforts for the creation of an IPD shared database, this amount of data for all the published schistosomiasis treatment trials of the past 15 years is disappointingly low, and reflects the lack of investments in clinical research in this aptly-named NTD.

### Conclusion

The present scoping exercise supports the interest of an antischistosomal efficacy data-sharing platform, as more than 20,000 IPD could contribute to extensive meta-analyses, using a common analytical plan. This would improve our knowledge of how effective treatments are in the overall population as well as in subgroups (age, location, etc.) and over time; and of how to analyse data in a meaningful way by exploring and comparing different approaches. The need for such a database is further emphasised by the push from funders and journals to make research data more widely accessible and optimally used. With this in mind, schistosomiasis stakeholders, researchers and policymakers have been invited to discuss their views on this matter at a WHO-convened meeting held in September 2015 [15]. Providing that a clear data governance structure is defined, especially regarding how data which are shared will be accessed and used, and that measures are taken to address the need for data management and analysis capacity-building, there was general support to the establishment of such a database towards producing a stronger evidence-base for schistosomiasis treatment.

## Supporting Information

S1 Checklist PRISMA checklist for the systematic review of antischistosomal trials. The template PRISMA checklist was downloaded from the PRISMA statement website and used to guide the reporting of our work. For each applicable item, we specify the sub-section or paragraph of this article where the information can be found.
(PDF)

S1 Dataset Source data supporting the systematic review of antischistosomal trials. The datasets (CSV files) contain the complete information extracted from the eligible articles or conference papers. An associated dictionary provides a detailed description of each dataset and each variable it comports. The scripts written to run the data analysis (using R 3.1.3) are also available.
(ZIP)

S1 Fig Time elapsed between the end of data collection and the dissemination of results. Represented data is for a total of 70 cohorts, as the date of completion of data collection was unknown for 34 cohorts. Each bar of the histogram represents the number of cohorts for which study outcomes were made publicly available (article publication or presentation at conference) within the corresponding 6-month period after the end of the data (biological samples) collection for that cohort. The red arrow points to the median reporting time (30 months = 2.5 years). The box covers values ranging between the first (18 months) and third (50 months) quartiles, whiskers extend to values up to 1.5 times the interquartile range.
(TIF)
