# Supplementary material for: The Schistosomiasis Clinical Trials Landscape: A Systematic Review of Antischistosomal Treatment Efficacy Studies and a Case for Sharing Individual Participant-Level Data (IPD)
Source: PLoS Negl Trop Dis. 2016 Jun 27;10(6):e0004784. doi: 10.1371/journal.pntd.0004784 (PMC4922574; doi:10.1371/journal.pntd.0004784)
Supplement: S1 Dataset — The datasets (CSV files) contain the complete information extracted from the eligible articles or conference papers. An associated dictionary provides a detailed description of each dataset and each variable it comports. The scripts written to run the data analysis (using R 3.1.3) are also available. (ZIP) [file pntd.0004784.s002.zip › sDB_varDictionary_final.pdf]

Research question(s)

- Is there scope for a data sharing platform for schistosomiasis treatment efficacy data, i.e. how many individual-level participant data (IPD) could be retrieved from past studies of anti-schistosomal?
- What are the characteristics of such studies conducted in the last 15 years?

Objectives and variables by dataset

0. Search Results

Full list of references identified through systematic search and explanations for inclusion/exclusion from subsequent analyses.

- Publication reference and source

**X00000**	Unique row identifier (X00010, X00020, etc.) given to the reference at entry into the database. This identifier X enables linkage between datasets.<br>NB: A reference may require several rows to capture information on all cohorts in other datasets (see 'Design' dataset for details), in which case children rows will be numbered X0001**1**, X0001**2**, etc.
**X0_ref**	Free text: Reference of the journal article or conference abstract, including all authors and full title. Formatting style: 'Elsevier Harvard (with titles)'.
**X1_source**	Factor variable, reflecting the source(s) of the reference (several may apply):<br>• "**published secondary analyses**" – reference found in the bibliography of at least one of the 5 previously published meta-/pooled analyses used as sources and manually screened for references;<br>• "**haematobium**, **mansoni** or **japonicum**" – reference found at search for studies on *S. haematobium* (respectively, *S. mansoni* and *S. japonicum*), performed on 27 May 2015;<br>• "**MFQ/OXA**" – reference found in the additional, separate search for studies on mefloquine and oxamniquine, performed on 12 June 2015.
**X2_level**	Factor variable, specifying if eligibility of the reference was assessed at the level of the "**full text**" (journal article or conference record) or using only a "**summary**" (abstract).
**X0_PMID**	PubMed ID, with hyperlink to the corresponding PubMed webpage.<br>Leave blank if the study was screened at the summary level only; enter "not found" if the full text was accessed but no corresponding PMID exists.

- Eligibility screening

**X2_screen**	Factor variable, describing reasons for excluding the reference before (pre-screen) or after (post-screen) detailed assessment of its content (see Appendix for details).
**X3_treat**	Dummy variable (**1** = Yes/**0** = No). The study involved the delivery of an antischistosomal drug (experimental or registered) to human participants. Of note, the study may have taken advantage of episode(s) of PCT/MDA for drug delivery to participants.<br>Interventions, other than drugs aiming at clearing the infection with *Schistosoma* (e.g. preventive *Schistosoma*-repellent cream, clinical management/surgery procedures, especially to mitigate symptoms), are out of scope (excluded at pre-screening).
**X4_pre (respectively, X4_post)**	Dummy variable (**1** = Yes/**0** = No). The study involved screening of participants for infection with a species of *Schistosoma* (*haematobium*, *japonicum* or *mansoni*) shortly before (resp., after) delivery of the intervention (baseline survey and individual follow-up, respectively). The screening procedure should involve a recognised diagnostic test (egg count in urine/stool, CCA).<br>Isolated surveys for prevalence monitoring, as well as studies which screen different sets of participants before and after treatment, are out of scope ("**0**").
**X5_inTime**	Dummy variable (**1** = Yes/**0** = No). At least one outcome assessment (post-treatment diagnostic test) occurred within 60 days (including, exactly 60 days or 2 months) after treatment, where administration of the first study drug counts as Day 0.
**X6_eligible**	Auto-filled variable, establishing whether the study is overall eligible. Equals **1** (= Yes) if and only if **X3_treat** + **X4_preTest** + **X4_postTest** + **X5_inTime** = 4 and **X2_screen** = "none"; otherwise, equals **0**.
**X7_dup**	Factor variable, listing references that seem to be duplicated reports of the same study. Reasons for excluding/including potential duplicates are discussed in **#notes** (see below).

- Has the reference been analysed and coded, and were issues encountered?

**X8_status**	Factor variable, capturing the progress of the review: "To be Analysed"; "**Analysed**"; "**Analysed (conference)**"; "**Excluded**"; "**Excluded (unclear)**"; "**Inaccessible**"; "**Inaccessible (unclear)**".
**#date**	Date (**DD/MM/YYYY**) of last update of the complete reading, analysis and coding of that reference.
**#notes**	Free text, 1 new line per dataset requiring comment(s).<br>Comments on problems encountered during analysis and coding of the article (missing or unclear data, impossibility to record the data in the desired format, inconsistency between different sources, etc.). Comments may also include details on reasons for inclusion/exclusion of studies whose eligibility was difficult to assess.<br>Leave blank if no comment is required.

1. Design

- Study identification

**A1_trialID**	Trial identifier, if the trial was registered and registration could be found (i.e. mentioned in the publication, or identified through search for 'schistosomiasis' + 'country of study' in ClinicalTrials.gov, Controlled-Trials.org and WHO ICTRP registries). Enter "not found" otherwise.<br>In alphabetical order of registry, and separated by semi-colon if several registries apply. Hyperlink to the most informative webpage, if available.
**A1_trial Registry**	Factor variable, corresponding to the name of the trial registry: "**ClinicalTrials.gov**"; "**Controlled-Trials.com (BioMed Central)**"; "**Pan African Clinical Trials Registry (PACTR)**". Enter "not found" otherwise.<br>In alphabetical order and separated by semi-colon if several registries apply.
**#studyTitle**	Scientific title of the study/trial, as stated at registration. Enter "not found" otherwise.
**#cohortsJustif**	Free text: justification for counting a single 'study' reported in this article/abstract as several 'cohorts' = set of participants sharing the same study meta-data characteristics (see Appendix for details).
**A0_S**	Unique study identifier (S1, S2, etc.), derived from the row identifier (X00010, X00020, etc.). If two or more rows are required to capture information on a same study (i.e. multiple cohorts), then they must have the same identifier A0_S.<br>NB: In the rare case where several studies are reported in the same publication, record them as S1a, S1b, S1c, etc.
**A0_C**	Unique cohort identifier (S1C1, S2C1, etc.) given as soon as a new cohort (set of participants) is added to the database. See **#cohortsJustif** and Appendix for details on breaking down studies into cohorts.

- What were the study objectives?

**A2_efficacy**	Dummy variable (**1** = Yes/**0** = No). Was the study designed primarily to assess efficacy or effectiveness of the treatment?<br>NB: Some studies may have been conducted for another purpose (e.g. diagnostic sensitivity assessment, immunology studies), in which case they score "**0**"; but they still collected data that would enable drug efficacy assessment in secondary analyses.
**A2_safety**	Dummy variable (**1** = Yes/**0** = No). Did the study assess the safety or tolerability of the treatment (i.e. collected data on side effects)?
**A2_pharma**	Dummy variable (**1** = Yes/**0** = No). Did the study involve some pharmacokinetics/pharmacodynamics (PK/PD) measures?
**A2_other**	Factor variable, describing other objectives of the study, namely:<br>• "**diagnostic approach**": diagnostic test study (e.g. sensitivity assessment), including search for biomarkers of infection which could potentially lead to new approaches to diagnostics;<br>• "**immunological responses**": assessment of specific Ab responses, etc.

	• "**nutrition**": impact of nutrient supplementation on treatment efficacy or other outcomes (e.g. performance at school).<br>If several levels apply, enter them in alphabetical order, separated by "+". Enter "none" otherwise.
**#objComment**	Free text: Additional comment/clarification on study type or aims, e.g. nested study, trial phase, significance for national programme. Enter "none" otherwise.

- How many arms did the study involve, including control-arm?

**A3_control**	Dummy variable (**1** = Yes/**0** = No). Was the intervention assessed against a comparator (or, did the study involve >1 arm)?<br>NB: Some studies are formally considered 'non-controlled' but they include a 'comparison' (e.g. of two age groups/settings), in which case they score "**1**".
**A3_ctrlCat1, 2**	Factor variable, describing the category of comparator used in controlled studies.<br>• "**drug comparison**"; "**dose comparison**"; "**regimen comparison**" – comparison of several antischistosomals against each other<br>• "**placebo**"; "**delayed treatment**"; "**untreated controls**"; "**healthy controls**"<br>• "**other** (**endemic context**, **season**, **treatment history**, or **education**)" – e.g. "other (endemic context)", for a comparison between low/high or new/old focus of transmission<br>If several levels apply, record them in the order suggested above. Enter "unclear" otherwise, or "N/A" for non-controlled studies and in superfluous columns.<br>NB: studies falling only into the category "other" are rather 'comparative' than 'controlled' studies (see **A3_control**).
**A3_arms**	Total number of arms in the study, including comparative groups if applicable.
**#armComment**	Free text: Description of the different arms, especially for unusual/complex designs. Enter "none" otherwise.

- Was the assignment to different arms randomised and blinded?

**A4_random**	Dummy variable (**1** = Yes/**0** = No). For multi-arms studies, was the assignment to one arm or the other random? If not explicitly specified, the assumption is that the study was not randomized.
**A4_rand Method**	Semi-free text: method of randomisation into the study if applicable.<br>(e.g. "**computer-generated sequence**"). Enter "unclear" otherwise, or "N/A" for non-randomized studies.
**A5_blind**	Dummy variable (**1** = Yes/**0** = No). For randomized studies, was the person delivering the drug and/or assessing the participant blinded to the intervention administered? If not explicitly specified or if drugs were distinguishable, the assumption is that the study was not blind.
**A5_blind Level**	Factor variable, describing the level of blinding used in the study if applicable: "**assessor-blinded**"; "**carer-blinded**"; "**double**"; "**triple**". Enter "unclear" otherwise, or "N/A" for non-blinded studies.

2. Setting

- Where was the study conducted/cohort recruited?

**B0_country**	Full name of the country of study. Use UN classification: http://unstats.un.org/unsd/methods/m49/m49regin.htm
**#siteLocation**	Free text: most precise name and address of each and every site of recruitment, including longitude and latitude GPS coordinates, if available.
**B1_siteCat**	Factor variable, describing the category of the site chosen as a recruitment centre of target participants: “**school**”; “**hospital/health-centre**”; “**other**” (village/city, households, etc.). Enter “unclear” otherwise.
**B1_setting**	Factor variable, describing the setting where recruited participants live: “**rural**”; “**semi-urban**”; “**urban**”. Use Google Earth if unclear in full text. If several apply, enter them in increasing level of urbanisation, separated by “+”. Enter “unclear” otherwise.
**B0_region**	World region of the country of study. Use UN classification: http://unstats.un.org/unsd/methods/m49/m49regin.htm
**B0_income**	Income group of the country – World Bank: http://data.worldbank.org/country
**B2_endem**	Factor variable, reflecting the prevalence of infection with *Schistosoma* in the study area – from “**0**” (prevalence of 0 to 9.99%) to “**9**” (90 to 99.99%), as found at screening (alternately, past estimates) based on egg count in excreta. Consider only the highest prevalence known for mixed endemicity contexts and/or if several sites are involved. If the prevalence is only qualitatively reported, enter “**0***” for ‘low’, “**4***” for ‘medium/intermediate’ and “**8***” for ‘high’ prevalence. Enter “-9” if unknown.
**B2_species1**	Factor variable, describing the *Schistosoma* species of interest at the study site.
**B2_species2**	Factor variable, describing the second *Schistosoma* species present at the study site, if applicable. If the prevalence of this second species among participants was checked only at baseline but not at follow-up, append “[partial data]”. If the prevalence of this second species was checked and found to be null, append “[absent]”, or possibly “[rare]”. Enter “N/A” otherwise.

- When was the study conducted and reported?

**B3_studyStart**	Start date of study (**DD/MM/YYYY**). If the exact date is unknown, then, arbitrarily, DD=15 and MM=03. Enter “unknown” otherwise.
**B4_studyEnd**	End date of study (**DD/MM/YYYY**). If the exact date is unknown, if possible, estimate it from start date and duration of follow-up, or, arbitrarily DD=15 and MM=09. Enter “unknown” otherwise.
**B5_reportDate**	Date of publication (earliest known, i.e. chose ‘ePub ahead of print’ date if available), or of presentation at a conference. If the exact day is unknown, then DD=15. If the exact month is unknown, chose ‘manuscript accepted’ date and add 1 month.
**B5_report Means**	Factor variable, describing the medium with which research results were reported: “**journal article**”; “**conference**”. NB: Only conference abstracts from the past year (2014-15) were analysed; it is assumed that studies presented at conference beforehand have been published by now and are therefore captured elsewhere.

3. Outcomes

- What (standard) analyses were performed to diagnose urinary schistosomiasis?

**#urinary Standard**	Semi-free text: description of the sample collection strategy and of the laboratory test performed to diagnose infection with *S. haematobium* – e.g. "2 to 3 samples (only 1 at baseline) collected between 10.00 am and 2.00 pm; 1 repeat (?); 10 mL filtration". Enter "none" if no diagnosis of urinary schistosomiasis by egg count was performed.
**C0_uSamp**	Minimum number of independent urine samples collected (and required for inclusion of in the study) to perform diagnosis at follow-up(s). Assume 1 if unclear; enter "N/A" if no test was performed.
**C1_uRep**	Minimum number of laboratory test repeats made on one same sample to perform diagnosis at follow-up(s). Assume 1 if unclear; enter "N/A" if no test was performed.

- What (standard) analyses were performed to diagnose intestinal schistosomiasis?

**#intestinal Standard**	Semi-free text: description of the sample collection strategy and of the laboratory test performed to diagnose infection with *S. mansoni/japonicum* – e.g. "aim for 3 samples but 2 tolerated; 2 repeats; Kato-Katz thick smears (cellophane, 50 mg)". Enter "none" if no diagnosis of intestinal schistosomiasis by egg count was performed.
**C0_iSamp**	Minimum number of independent stool samples collected (and required for inclusion in the study) to perform diagnosis at follow-up(s). Assume 1 if unclear; enter "N/A" if no test was performed.
**C1_iRep**	Minimum number of laboratory test repeats made on one same sample to perform diagnosis at follow-up(s). Assume 1 if unclear; enter "N/A" if no test was performed.
**C3_iTech1, 2**	Factor variable, describing the technique employed to analyse stools. "**Kato-Katz thick smears (XX mg)**"; "**Kato-Katz method** (= technique, test)"; "**Kato-Katz smears (modified)**"; "**Katz thick smears**"; "**Kato thick smears (XX mg)**"; "**Kato technique (qualitative)**"; "**concentration (formaldehyde)**"; "**hatching test**"; "other". Information in brackets may or not be recorded depending on it being detailed in the full text or not. If several apply, use additional columns and record in the order suggested above. Enter "N/A" if no test was performed and in superfluous columns.

- What other tests were performed to diagnose and characterise schistosomiasis, as well as other biomedical assays (thus providing additional, secondary outcome measures)?

**C4_testClinic**	Semi-free text: Additional point-of-care (POC)/rapid test or clinical assessment to diagnose and characterise schistosomiasis (including symptoms); mainly biopsy, dipsticks/reagent strips for haematuria, proteinuria or leukocyturia, visual aspect of excreta, POC circulating antigen, ultrasonography. Enter "none" otherwise.
**C4_testLab**	Semi-free text: Additional molecular biology assay performed to diagnose and characterise schistosomiasis, mainly ELISA and PCR. Precise the source of biological material (blood, stool, urine…), and the type of molecules assessed in molecular assays (e.g. *Schistosoma* DNA, soluble egg Ag…). Enter "none" otherwise.

**C5_other Assays**	Factor variable, describing other biomedical assays: "**Hb concentration**"; "**other serology/immunology assays**" (serology for HIV/AIDS diagnosis, cytokines' profile after PBMC culture, etc.); "**other blood analyses**" (e.g. haematocrit, iron absorption).

- When were participants followed-up for outcome assessment(s)?

**D1_T1, 2, 3, last**	Time of first (respectively, second, third or latest) follow-up, in days, counted from the day of first treatment dose (Day 0), up to the day of first re-sampling. If the time is specified in weeks, months, or years in the publication, use 1 week = 7 days, 1 month = 30 days and 1 year = 365 days to convert. Enter "none" if only one (resp. 2 or 3) time point(s) applies.

- How was the primary, drug efficacy endpoint expressed?

**D3_CR**	Dummy variable (**1** = Yes/**0** = No). Was the cure rate (CR) calculated at any time of follow-up? CR: percentage of participants cured (i.e. excreting no egg) at follow-up, among participants with confirmed infection (i.e. excreting eggs) at baseline.
**D4_ERR**	Dummy variable (**1** = Yes/**0** = No). Was the egg reduction rate (ERR) calculated at any time of follow-up? ERR: difference between mean egg count at follow-up and at baseline, among participants with confirmed infection.
**D4_intens**	Dummy variable (**1** = Yes/**0** = No). Was there a quantitative comparison of pre- and post-treatment intensity of infection? This variable is particularly relevant for studies which do not report the ERR, but still report and compare the intensity of infection before <u>and</u> after treatment as a mean, median and/or other quantitative measure (i.e. not only qualifying the intensity as 'low' or 'heavy').
**D5_eggsMean**	Factor variable, describing the population mean utilised to calculate the ERR: "**arithmetic**"; "**geometric**"; "**both**". Enter "unclear" otherwise, or "N/A" if no ERR was calculated.
**#endpoint Comment**	Free text: Specifications on other times of follow-up that could not be recorded in dedicated columns ("**Additional time-points**"), on the way the outcome was assessed ("**Limits**" – e.g. time-point applicable to a subset of participants only, efficacy endpoint derived from other measure than egg count), or additional notes ("**NB**").

4. Participants

- Number of participants and type of schistosomiasis infection that they were required to have for inclusion in the study

**D0_included**	Total number of participants in the initial cohort; i.e. who were *included into the study and received the intended intervention* (drug(s), control, placebo, no-treatment, etc.), with the intention to follow them up for outcome assessment. Enter "-9" if unknown.
**E0_infection**	Factor variable, describing the infection with which species that was necessary *to be included in the study and to receive the antischistosomal treatment* (do not consider participants infected with other parasites who were specifically treated against those): "**Sh+**"; "**Sj+**"; "**Sm+**". Enter "not required" otherwise.<br>If several species apply, precise into brackets which confirmed infection was "**required**" for inclusion; and which co-infection was either "**tolerated**" or "**excluded**" among study participants. Record several species in the order suggested above ("required", "tolerated", "excluded" infection), and then in alphabetical order of species. Separate different levels by "**and**" (if both infections were concomitantly required)/"**or**" (if one or the other infection was required), accordingly – e.g. "Sm+ (required) and Sh+ (tolerated)"; "Sh+ and/or Sm+".
**D1_infected**	Number of participants whose individual data would enable assessment of drug efficacy; i.e. participants who *were infected with Schistosoma spp. at baseline and received an antischistosomal drug or comparator, with the intention to follow them up* for outcome assessment within 60 days post-treatment. In some cases (especially, studies in which individuals non-infected with *Schistosoma* spp. at baseline were still included in the study), this exact number of participants is unknown, and a conservative estimate should be calculated – i.e. total included in analyses (**D0_included**) x Prevalence in area. If no correction is needed, re-enter **D0_included**. Enter "-9" if unknown.
**D2_followed**	Number of relevant participants (i.e. among **D1_infected**) who were followed-up. If follow-up is detailed for each of the different time-points, enter the maximum number of participants followed-up. If the exact number is unknown (i.e. for studies in which individuals non-infected with *Schistosoma* spp. at baseline were still included in analyses), use the same correction factor as was used to obtain **D1_infected** from **D0_included**. Enter "-1" if not applicable (e.g. when 'provide samples at follow-up' is an inclusion criterion, meaning that the study cohort was defined *a posteriori*, based on compliance); and "-9" if unknown.
**#corrected Calculation**	Free text: way in which the infected number of participants (**D0_infected**) was obtained from the total of included participants (**D0_included**), if applicable. Enter "N/A" otherwise.
**E1_schisto Include**	Free text: other criteria related to schistosomiasis and that were applied to include participants – e.g. minimum number of eggs required. Enter "none" otherwise.
**E1_schisto Exclude**	Free text: other criteria related to schistosomiasis and that were applied to exclude participants – e.g. symptomatic form of the disease.

- What major (non-*Schistosoma*) co-infections were controlled for, and possibly resulted in exclusion from the study?

**E2_malaria**	Dummy variable (**1** = Yes/**0** = No). Was co-infection with malaria assessed (at least at baseline) using a diagnostic test?
**E2_excluMal**	Dummy variable (**1** = Yes/**0** = No). Did infection with malaria (as confirmed by diagnostic test or clinical assessment only) result in exclusion from the study?
**E3_helminth**	Dummy variable (**1** = Yes/**0** = No). Was co-infection with at least 1 other helminth assessed (at least at baseline) using a diagnostic test?
**F3_excluHelm**	Dummy variable (**1** = Yes/**0** = No). Did infection with at least 1 other helminth result in exclusion from the study?

- What is the age range of enrolled participants and could they be pregnant?

**F0_ageMin (resp. Max)**	Minimum (resp. Maximum) age, in years, among enrolled participants. Strictly speaking, this does not necessarily correspond to the age range specified in inclusion/exclusion criteria for the study. Enter "-9" otherwise.
**F5_pregnancy**	Semi-free text, stating first if pregnant women were "exclude[d]" or "include[d]", and how pregnancy was defined (e.g. "exclude: pregnant or lactating women, pregnancy test"). Enter "N/A" if age range (<12 years old) suggests no pregnant female could have been included; enter "unclear" otherwise.

- Specific inclusion criteria[1]

**G0_inclu Healthy**	Free text: general health status required for inclusion, along with criteria for judgement (e.g. "appear healthy at enrolment, as assessed by the study physician", "no diarrhoea").
**G1_incluMorph**	Semi-free text: weight and/or height limits for inclusion (e.g. "weight > 25 kg").
**G3_inclu Compliant**	Free text: ability and willingness to follow/minimal compliance to the protocol required for inclusion, especially in analyses (e.g. "able and willing to be examined by the study physician at baseline and follow-up").
**G4_inclu Consent**	Free text: method for securing informed consent, including who gave consent for children (parent, head of the school, etc.) and how (orally, in writing, etc.).
**G5_inclu School**	Free text: school grades among which children were recruited, and/or other criteria for inclusion related to the school (e.g. "be present at school on screening day" or "school accessible by road during rainy season").
**G6_inclu Residency**	Free text: duration of residency in the study area, and/or other criteria of inclusion related to the area of residence (e.g. "living in the study area and intending to deliver at the Entebbe Hospital" or "residing in the study area since birth").

[1] When inconsistency between sources (e.g. trial registry and journal article), report most stringent criteria; unless additional information permits to say which source is accurate.

**G7_incluOther**	Free text: other inclusion criteria not falling into any of the previous categories.

- Specific exclusion criteria[2]

**H0_exclu Malnutrition**	Free text: state of malnutrition and/or anaemia leading to exclusion, along with decision rule if available (e.g. "signs of micronutrient deficiencies" or "signs of severe malnutrition (defined as children with weight/height ratio <3 SD or <70% of the median of WHO standardised reference values, or still with symmetrical oedema affecting both feet)").
**H3_excluIllness**	Free text: list of severe illnesses or systematic diseases leading to exclusion.
**H4_exclu Allergy**	Free text: precisions on allergies leading to exclusion, essentially hypersensitivity to study drugs.
**H5_exclu Medication**	Free text: medication(s) taken during a specific period prior to (or during) the study and leading to exclusion, essentially any antischistosomal within the past 28 days.
**H7_exclu Participation**	Free text: multiple participation in the same or similar studies, or concomitant participation in another trial leading to exclusion.
**H8_excluOther**	Free text: other exclusion criteria not falling into any of the previous categories.

[2] When inconsistency between sources (e.g. trial registry and journal article), report most stringent criteria; unless additional information permits to say which source is accurate.

5. Treatment

- Treatment regimen and corresponding number of participants in group 1, 2, etc.

**#detail Regimen1, 2, etc.**	Free text: exact names and doses of drugs assigned to group 1. Provide details on how the number of participants *treated* with that regimen was obtained, if unclear (e.g. for studies in which individuals non-infected with *Schistosoma* spp. at baseline were still given treatment). Enter “none” in superfluous columns.<br>NB: For the purpose of this descriptive review, participants who received an antischistosomal (standalone) or the same antischistosomal together with a placebo are considered within one same group.
**I0_regCat1, 2, etc.**	Factor variable, describing the category of the treatment regimen. If several factors apply, enter them in the order suggested below, separated by “+”.<br>• “**PZQ (XX mg/kg)**”: praziquantel and corresponding dose in mg/kg body weight – where the dose corresponds to the *overall dose received within 1 week*<br>• “**antischistosomal (other)**”: any non-PZQ, antischistosomal drug(s)<br>• “**anthelmintic (other)**”: any non-antischistosomal, used against other helminths<br>• “**ACT**”: Artemisinin-based Combination Therapy<br>• “**antimalarial (other)**”: any non-ACT, antimalarial drug(s)<br>• “**control**” or “**control (healthy)**”: placebo or no treatment, and possibly uninfected at baseline<br>• “**other**”: any drug(s) not falling in one of the previous categories<br>Enter “N/A” otherwise, i.e. in superfluous columns.
**I1_reg1, 2, etc.<br>I2_reg1, 2, etc.<br>I3_reg1, 2, etc.**	Abbreviated name of the first (**I1_regX**), second (**I2_regX**) and third (**I3_regX**) drug(s) given as part of the assigned regimen. Record drugs in the order suggested below. Drugs in brackets may have been given to some participants in that group, but not all (e.g. albendazole, if infected with other helminths; placebo or not in addition to an interventional drug). Enter “N/A” in superfluous columns.<br>• Dose of praziquantel, recorded in the format “**(NxQQ):Tu**”, where:<br>▪ N = total number of split doses received within the study. Add a star (*) if this dose is a repeat of a past treatment, or will be possibly repeated for a subset of participants, e.g. at third follow-up if treatment failure.<br>▪ QQ = quantity (in mg/kg) per split dose administered.<br>▪ Tu = time T (and its unit u) that separated 2 split doses. If several times were possible (e.g. every 4 to 6 hours), enter the shortest interval.<br>• “**OXA**” = Oxamniquine; “**myrrh**” = Mirazid<br>• “**As**” = Artesunate; “**AM**” = Artemether<br>• “**A**” = Amodiaquine; “**MFQ**” = Mefloquine<br>• “**SP**” = Sulfadoxine-Pyrimethamine; “**SMP**” = Sulfamethoxypyrazine-Pyrimethamine; “**LU**” = Lumefantrine<br>• “**TCBZ**” = Triclabendazole; “**ALB**” = Albendazole; “**placebo**”; “**none**”.
**I4_treated1, 2, etc.**	Number of participants infected at baseline and who fully received the treatment they were assigned. If the number of participants successfully treated is not specified, enter the number of *participants initially assigned to that arm* – i.e. assuming they all completed the treatment. When several doses of the same drug were possibly repeated, the number recorded is the number of participants who received at least 1 dose of the treatment, if known – i.e. maximum number treated with any number of doses. Enter “-9” if unknown, and “-1” if not applicable (superfluous columns).

Appendix

Detailed explanation of "**X2_screen**" variable and decision-tree for study eligibility assessment

Some references can be excluded quickly, before even reading their full summary. The "pre-screens" are successive 'checkpoints', which a reference must all go through before progressing to further assessment.

- "**Pre-screen 0: conference prior 2014**" – excluded because 'too old', as judged from the dates of the conference
- "**Pre-screen 1: case report**" – excluded because it reports on a single or series of case(s), but does not involve execution of a pre-planned clinical study
- "**Pre-screen 2: review**" – excluded because it is a review of the literature (textbook chapters, scoping or systematic review, meta-analyses, etc.), or a comment/opinion paper, but does not involve primary data collection
- "**Pre-screen 3: in vitro/ex vivo, laboratory work, no human subject**" – excluded because the study is performed in the laboratory and/or does not involve human subjects
- "**Pre-screen 4: no *Schistosoma spp.* infection, no antischistosomal drug delivery, non-endemic setting**" – excluded because the study is not on *Schistosoma spp.* (no participant assessed for this infection), or the intervention delivered is not aiming to clear infection with this parasite, or the study is not performed in the endemic setting where infection occurred

When progressing to more detailed assessment, excluded studies are categorised in one of 6 broad "topics" to clarify the reason for their exclusion.

- "**Topic 0: individual patient data for drug efficacy, with late outcome assessment**" – fulfils all inclusion criteria, except that the outcome measure was taken too late (>60 days post-treatment);
- "**Topic 1: associated morbidity and immunological responses, co-infections, risk factors**" – IPD would not enable antischistosomal efficacy assessment; the study focusses on symptoms, co-infections and morbidity associated to schistosomiasis, or on immunological responses to infection and/or treatment;
- "**Topic 2: diagnostic approach, prevalence estimation method**" – IPD would not enable antischistosomal efficacy assessment; the study focusses on development of new diagnostic assays, or on optimised use of existing tests into the field (e.g. for epidemiological mapping);
- "**Topic 3: behaviour, education, qualitative research, implementation piloting and safety**" – did not involve diagnostic testing of participants, as the focus is on the safety/accuracy of programme delivery (e.g. multiple drugs within national preventive chemotherapy, dose assessed using height as a proxy for weight), or on behavioural/educational factors associated to (re-)infection;
- "**Topic 4: cost-effectiveness, modelling, projections and simulations *in silico***" – did not focus on treatment efficacy data collection into the field, but on mathematical modelling (using past, secondary data or not).

- "**Topic 5: endemicity, disease mapping, surveillance, epidemiological survey**" – did not involve a pre-/post-treatment diagnostic assessment (or at least not at the individual level), as the focus is on prevalence estimation and monitoring.

Finally, some studies are excluded after detailed assessment, and reason for their late exclusion is explained by the "post-screens".

- "**Post-screen 1: study end prior 2000**" – although published within the chosen timeframe, the study is too 'old', i.e. completed before 1st January 2000
- "**Post-screen 2: redundant, several references reporting on same study**" – several articles reporting on the same study were identified, and only the most exhaustive one was included

If no reason was found to exclude the reference, enter "none". If the situation is still unclear (e.g. due to inaccessible full text), enter "unknown".

Reasons for considering a single study as several cohorts

A study is to be divided accordingly into different cohorts if and only if at least one of the four following conditions apply:

1. Multi-centric study, with centres in several countries → 1 cohort per country
2. Multi-centric study, with centres possibly in the same country but infested with different *Schistosoma* species (including mono- *vs.* mixed endemicity sites) → 1 cohort per endemic setting
3. The study clearly differentiates *a priori* between different categories of participants, in particular in terms of age group (e.g. mothers/children, infants/school-aged children) → 1 cohort per participant group
4. The protocol is otherwise amended depending on site/group (e.g. different diagnostic approach, different follow-up time-points) → 1 cohort per protocol
