# Supplementary figures and images for: The Schistosomiasis Clinical Trials Landscape: A Systematic Review of Antischistosomal Treatment Efficacy Studies and a Case for Sharing Individual Participant-Level Data (IPD)
Source: PLoS Negl Trop Dis. 2016 Jun 27;10(6):e0004784. doi: 10.1371/journal.pntd.0004784 (PMC4922574; doi:10.1371/journal.pntd.0004784)

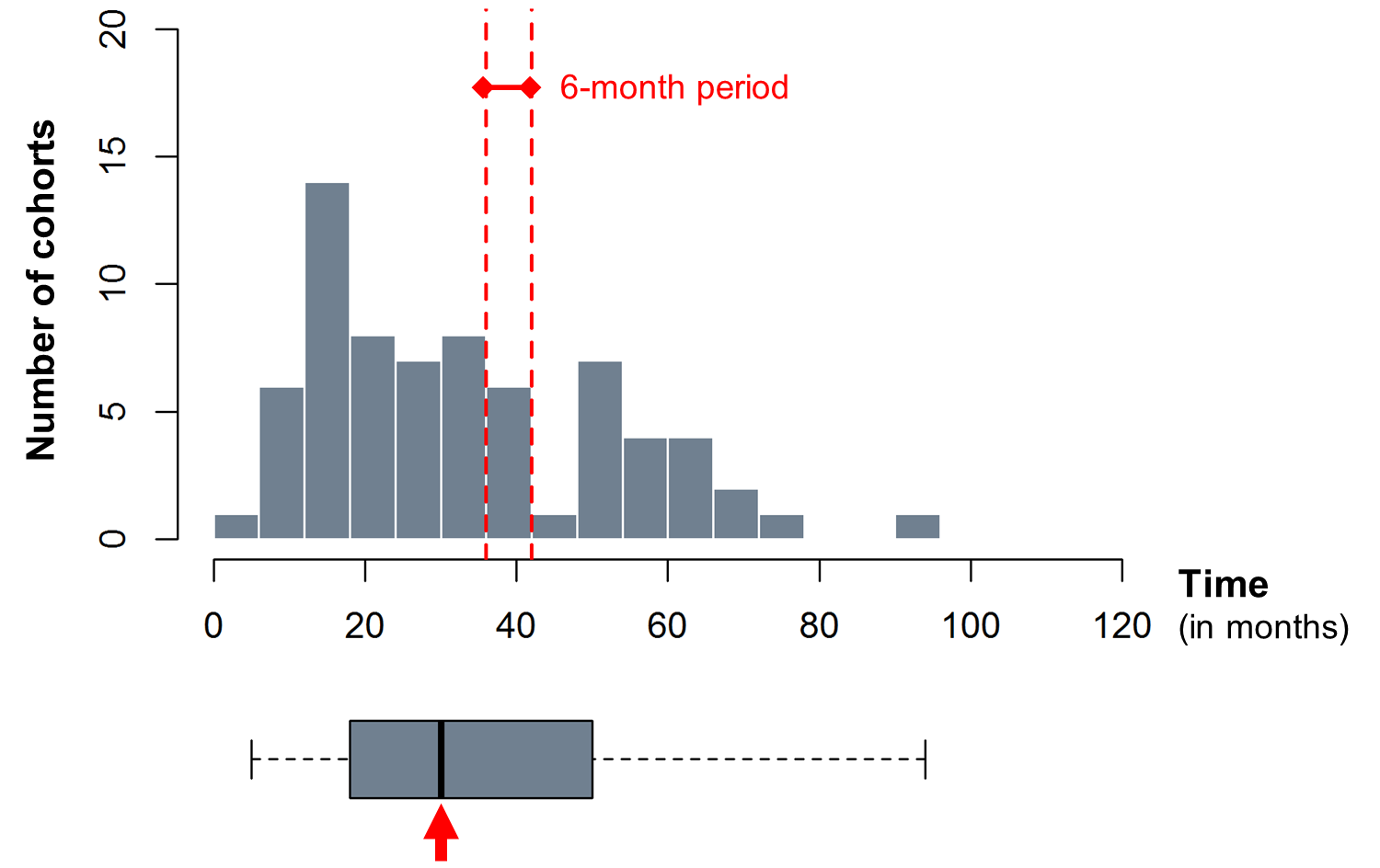

Supplement: S1 Fig — Represented data is for a total of 70 cohorts, as the date of completion of data collection was unknown for 34 cohorts. Each bar of the histogram represents the number of cohorts for which study outcomes were made publicly available (article publication or presentation at conference) within the corresponding 6-month period after the end of the data (biological samples) collection for that cohort. The red arrow points to the median reporting time (30 months = 2.5 years). The box covers values ranging between the first (18 months) and third (50 months) quartiles, whiskers extend to values up to 1.5 times the interquartile range. (TIF) [file pntd.0004784.s003.tif]
